# Supplementary material for: Effect of Clay Nanofillers on the Mechanical and Water Vapor Permeability Properties of Xylan–Alginate Films
Source: Polymers (Basel). 2020 Oct 4;12(10):2279. doi: 10.3390/polym12102279 (PMC7601507; doi:10.3390/polym12102279)
Supplement: Supplementary file 1 [file polymers-12-02279-s001.pdf]

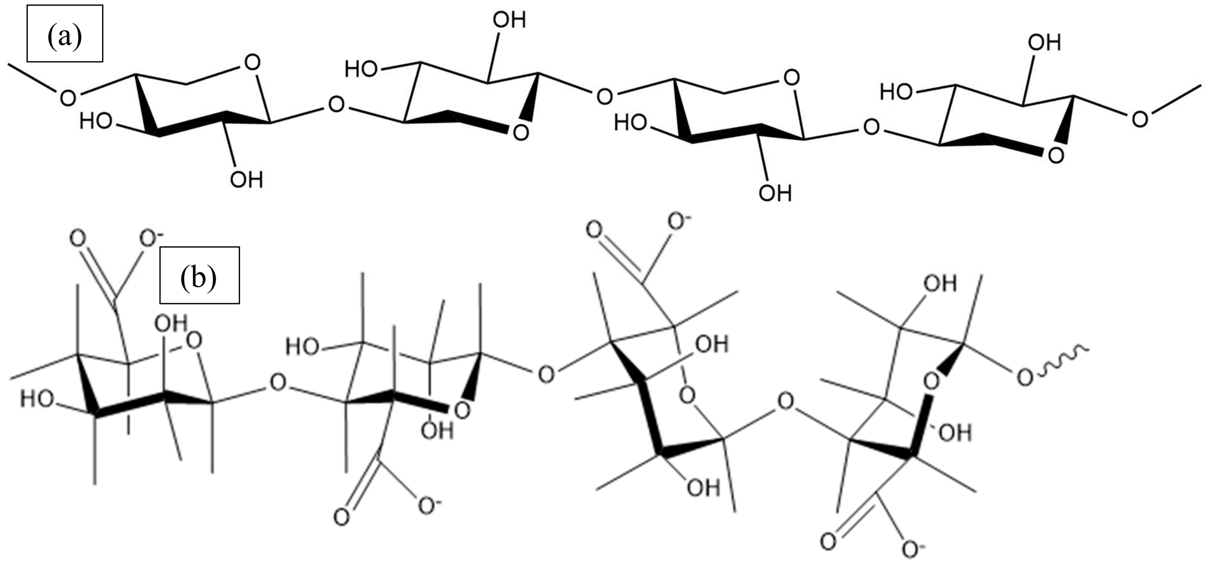

Figure S1: Chemical structure of (a) homoxylan and (b) alginate.

Table S1: Surface area and pore size of clay used as reinforcement.

Clay	BET surafce area/ $m^2.g^{-1}$	Pore size/ Å
Bentonite	20.42	59.00
Halloysite	26.35	57.47

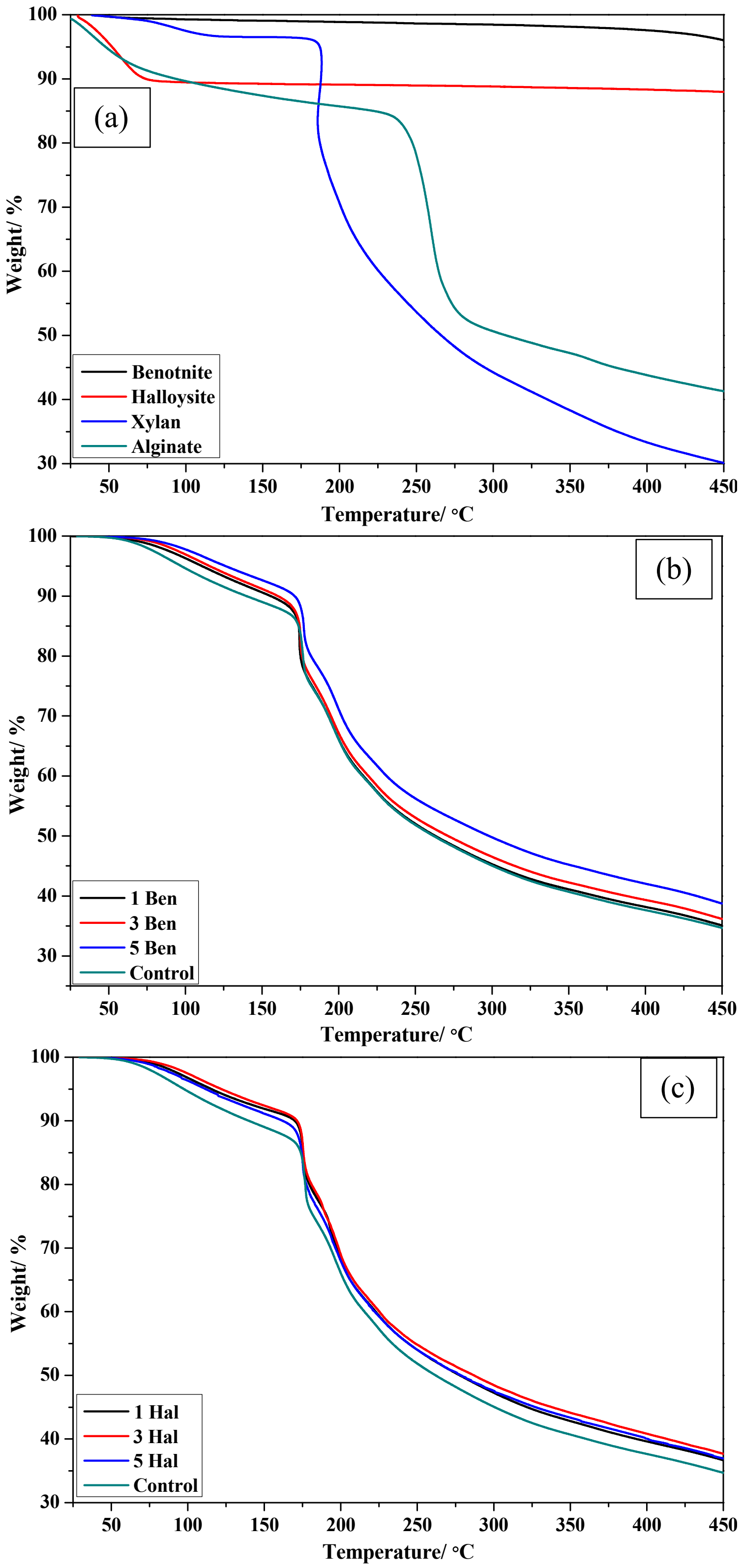

Figure S2: Thermograms of (a) xylan, alginate, bentonite and halloysite, (b) xylan-alginate films containing bentonite and (c) xylan-alginate films containing halloysite.

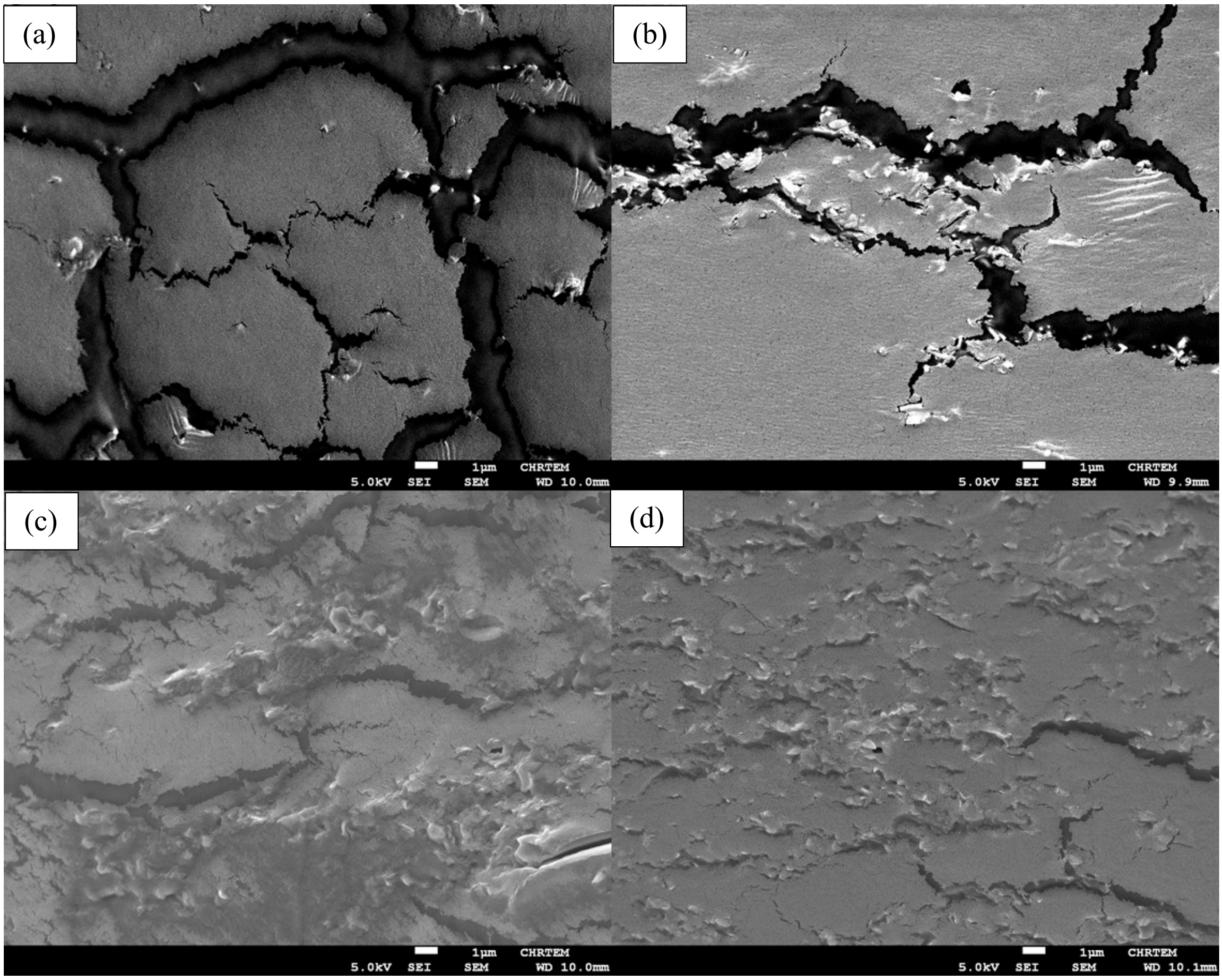

Figure S3: SEM cross-section images of films containing (a) 1wt% halloysite, (b) 3wt% halloysite, (c) 1wt% bentonite and (d) 3wt% bentonite.
